# Characterization of Total RNA, CD44, FASN, and PTEN mRNAs from Extracellular Vesicles as Biomarkers in Gastric Cancer Patients

**DOI:** 10.3390/cancers13235975

**Published:** 2021-11-27

**Authors:** Philipp Rhode, Matthias Mehdorn, Orestis Lyros, Christoph Kahlert, Thomas Kurth, Tom Venus, Katrin Schierle, Irina Estrela-Lopis, Boris Jansen-Winkeln, Florian Lordick, Ines Gockel, René Thieme

**Affiliations:** 1 Department of Visceral, Transplant, Thoracic and Vascular Surgery, University Hospital Leipzig, D-04103 Leipzig, Germany; philipp.rhode@medizin.uni-leipzig.de (P.R.); matthias.mehdorn@medizin.uni-leipzig.de (M.M.); orestis.lyros@medizin.uni-leipzig.de (O.L.); boris.jansen-winkeln@sanktgeorg.de (B.J.-W.); ines.gockel@medizin.uni-leipzig.de (I.G.); 2 Department for Visceral, Thoracic and Vascular Surgery, University Hospital Carl Gustav Carus, D-01307 Dresden, Germany; christoph.kahlert@uniklinikum-dresden.de; 3 Center for Molecular and Cellular Bioengineering (CMCB), Technology Platform, Technische Universität Dresden, D-01307 Dresden, Germany; thomas.kurth@tu-dresden.de; 4 Institute of Medical Physics and Biophysics, University of Leipzig, D-0407 Leipzig, Germany; tom.venus@medizin.uni-leipzig.de (T.V.); irina.estrela-lopis@medizin.uni-leipzig.de (I.E.-L.); 5 Institute of Pathology, University Hospital Leipzig, D-04103 Leipzig, Germany; katrin.schierle@medizin.uni-leipzig.de; 6 Department of Oncology, Gastroenterology, Hepatology, Pulmonology and Infectious Diseases, University Hospital Leipzig, D-04103 Leipzig, Germany; florian.lordick@medizin.uni-leipzig.de; 7 University Cancer Center Leipzig (UCCL), University Hospital Leipzig, D-04103 Leipzig, Germany

**Keywords:** extracellular vesicle (EV), mRNA, gastric cancer, fatty acid synthase (FASN)

## Abstract

**Simple Summary:**

Liquid biopsy is an easily accessible and non-invasive method to gain information about tumor diseases. The purpose of our study was to determine the value of extracellular vesicle-derived mRNAs as biomarkers for the diagnosis of gastric cancer and the response to its treatment. In a cohort of 87 gastric cancer patients and a control group of 14 individuals, we analyzed the absolute RNA concentration from extracellular vesicles (EV) and the relative levels of FASN, PTEN, and CD44 mRNA, and their correlation with clinico-pathological features. These correlated with treatment, tumor grading, and the pathological subtype according to Laurén’s classification. This might reflect their potential as both diagnostic and therapeutic predictors.

**Abstract:**

In-depth characterization has introduced new molecular subtypes of gastric cancer (GC). To identify these, new approaches and techniques are required. Liquid biopsies are trendsetting and provide an easy and feasible method to identify and to monitor GC patients. In a prospective cohort of 87 GC patients, extracellular vesicles (EVs) were isolated from 250 µL of plasma. The total RNA was isolated with TRIZOL. The total RNA amount and the relative mRNA levels of CD44, PTEN, and FASN were measured by qRT-PCR. The isolation of EVs and their contained mRNA was possible in all 87 samples investigated. The relative mRNA levels of PTEN were higher in patients already treated by chemotherapy than in chemo-naïve patients. In patients who had undergone neoadjuvant chemotherapy followed by gastrectomy, a decrease in the total RNA amount was observed after neoadjuvant chemotherapy and gastrectomy, while FASN and CD44 mRNA levels decreased only after gastrectomy. The amount of RNA and the relative mRNA levels of FASN and CD44 in EVs were affected more significantly by chemotherapy and gastrectomy than by chemotherapy alone. Therefore, they are a potential biomarker for monitoring treatment response. Future analyses are needed to identify GC-specific key RNAs in EVs, which could be used for the diagnosis of gastric cancer patients in order to determine their molecular subtype and to accompany the therapeutic response.

## 1. Introduction

Gastric cancer (GC) is the fifth most common cancer entity worldwide. There are over 1 million new diagnosed cases and 748,000 GC-related death per year. Despite multi-modal therapeutic approaches, cancer-related mortality from GC is high [1]. While gastric cancer is diagnosed early due to available screening programs in Asia (e.g., Japan and South Korea), diagnoses in western countries are mostly late with locally advanced tumor stages [2]. However, the incidence in Asian countries is much higher than in western populations [3]. In Europe, the standard of care for the treatment of locally advanced GC is perioperative chemotherapy with surgery [4,5,6]. Perioperative chemotherapy is recommended for all tumor categories ≥ cT3 or ≥cN1 [7]. Most clinical studies allow the perioperative chemotherapy from UICC stage: ≥Ib (T2 N0 M0 und T1 N1 M0). Locally advanced adenocarcinomas of the esophagogastric junction (EGJ) are treated similarly GC with a perioperative chemotherapy; alternatively, a neoadjuvant chemoradiotherapy can be applied [8]. Currently, the median survival rate could be improved by 15 months from 35 to 50 months using the FLOT (5-FU, leucovorin, oxaliplatin, docetaxel) regimen as perioperative chemotherapy [9]. Approximately 50% of patients progress to incurable metastatic disease despite multimodal therapy. So far, no biomarkers are available or validated to predict chemotherapy response in GC patients. The decision for perioperative chemotherapy is mainly based on tumor stage (computer tomographic (CT)-staging and endosonography), regardless of the tumor biology. Currently, there is only one molecular marker, HER2 (Epidermal Growth Factor Receptor 2), available for GC patients, which is routinely analyzed in clinical practice [10]. However, promising targetable molecules such as PD-L1, PD-1, VEGF, Claudin-18.2, and FGFR2b are currently under investigation, to discover more target-orientated treatment options for GC [8]. Recently, additional molecular subtypes have been introduced to supplement the histo-pathological classification according to Laurén and the WHO [11,12]. Thereby, dysregulated pathways have been identified, and a robust molecular classification has been proposed with four distinct subclasses of GC. The molecular subtypes should help to stratify patients and to guide them to trials of further targeted therapies [11]. When considering GC patients with regard to their molecular subtypes, they correlate with the overall survival (OS), with the best OS in patients in the Ebstein–Barr virus (EBV)-related subtype and the worst in patients with a genomic stable (GS) subtype [13]. The surface marker CD44 is involved in cell motility and proliferation. CD44 is known to be amplified in GC and is part of the molecular stratification supposed by “The Cancer Genome Atlas” (TCGA) program [11]. Additionally, CD44-positive GCs have been associated with lymphatic and distant metastases [14]. The tumor suppressor PTEN, which is often deleted in GC, antagonizes the PI3K/Akt-signaling pathway to induce apoptosis and restricts cellular differentiation [11,15]. PTEN-negative GCs are associated with metastasis and tumor invasion [16]. The fatty acid synthase (FASN) is overexpressed in many tumor entities because of tumor cells’ dependency on the “de novo” synthesis of fatty acids compared to normal healthy tissues [17]. GC patients that are positive for FASN had higher tumor stages and a higher risk of distant metastasis [17]. However, an easy and reliable screening tool, complementary to endoscopic GC appraisal, is desirable, and a molecular profiling of tumor material is invasive, time consuming and expensive. Therefore, liquid biopsies have the capacity to provide biomarkers for GC patients. Alongside the classical tumor markers, e.g., CEA, CA 19-9 or CA 72-4, liquid biopsies contain cell-free DNA (cfDNA), extracellular vesicles (EV, e.g., exosomes), free microRNAs, and circulating tumor cells (CTCs) [18].

EVs, such as exosomes, are secreted, membrane-enclosed vesicles with a diameter of 50–150 nm [19]. Released from cells, EVs contain nucleic acids, proteins, and cell plasma components [20,21], while members of the tetraspanin family (CD9, CD63, and CD81) are enriched at exosomes’ surface. Furthermore, endosomal sorting complexes (TSG101 and Alix) and heat-shock proteins (Hsp60, Hsp70, and Hsp90) are abundant in exosomes [22]. EVs contain different cargos, such as nucleic acids (DNA, mRNA, microRNA, long non-coding RNA) [21,23,24], proteins [25,26], lipids [27], and metabolites [28]. There is also increasing evidence that exosomes mediate cell–cell communication and transfer biologically active cargos [29,30,31]. Distinguishing non-cancer EVs from cancer cell-derived EVs will guide the diagnosis of early cancer and treatment control. No reliable EV biomarkers are available for GC patients yet. However, a recent study identified syntenin-1 as putative universal exosomal biomarker by quantitative proteomics [32]. A position statement for the pre-processing and pre-analytical properties, EV separation and analyses are given by International Society for Extracellular Vesicles [33].

CD44, PTEN, and FASN were chosen exemplarily to clarify the feasibility of the determination of mRNA levels from EVs and their clinico-pathological correlation in GC patients. In regard to the TCGA analyses, CD44 was shown to be amplified, while PTEN is often deleted in GC [11]. FASN overexpression was correlated with higher tumor stages and a higher risk for metastases in GC [17]. Here, we could demonstrate the feasibility of total RNA isolation from EVs and show the dependency of total RNA amount, CD44, and FASN mRNA-levels with regard to patients’ clinical treatment course. The usage of mRNA profiles from EVs might be useful to stratify patients for their molecular subtype and to use EVs as potential biomarkers for disease progression and therapeutic response (chemo/chemoradiotherypy, biologicals).

## 2. Materials and Methods

### 2.1. Patients

In this single center study, samples were consecutively collected and retrospectively analyzed. Patients with histologically confirmed GC, age ≥ 18 years and not suffering from a second malignancy were eligible for this study. The monocentric study was performed, comprising a total of 87 patients. Additionally, a control group of 14 patients with Barrett’s metaplasia was included. Patients with Barrett’s metaplasia had undergone an esophagogastroduodenoscopy at the time point of blood sampling and were confirmed not to be suffering from an upper GI tumor. Patients gave their informed and written consent to participate in this study. The study is in accordance with the declaration of Helsinki, and the protocol was approved by the local ethics committee of the University of Leipzig (No. of the approval: 038-16 and 106-16). The TNM categories and the UICC stages were determined according to the 8th edition of the TNM classification of malignant tumors. Unfortunately, patients in palliative situations did not receive a complete TNM staging. The pathological response rate (PRR) was determined in specimens from patients that had undergone surgery. An expert GI pathologist (K.S.) evaluated the PRR according to the method of Werner and Höfler [34]. The classification is based on the percentage of viable tumor cells after neoadjuvant therapy (grade 1: complete response; grade 2: partial response; grade 3: weak or no response).

### 2.2. EV Isolation from Plasma

Venous blood was collected by an S-Monovette^®^ (K3 EDTA, Sarstedt, Nümbrecht, Germany). The blood was centrifuged twice (400× *g* at 4 °C for 5 min and 15,000× *g* at 4 °C for 10 min) to obtain plasma and stored at −20 °C. EVs were isolated by the “Total Exosome Isolation Kit” from Plasma (ThermoFisher, Darmstadt, Germany) according to the manufacturer’s protocol. Briefly, EVs were isolated from 250 µL of plasma by adding 125 µL of PBS and 12.5 µL of proteinase K (20 mg/mL), vortexed and incubated at 37 °C for 10 min. Afterwards, 75 µL of EVs precipitation reagent was added, vortexed and incubated at 4 °C for 30 min. The precipitated EVs were pelleted by centrifugation (10,000× *g* at 4 °C for 10 min), and the supernatant was discarded.

### 2.3. Transmission Electron Microscopy (TEM)

Isolated EVs were dissolved in PBS, placed onto a 400 mesh carbon/formvar-coated grids and absorbed for a minimum of 1 min. Afterwards, the grids were washed with PBS and distilled water, allowed to dry and counterstained with 1% aqueous uranyl acetate. TEM was performed with a Jeol JEM1400Plus transmission electron microscope (Jeol, Fresing, Germany), equipped with a Ruby Camera (Jeol, Freising, Germany), and run at 80 kV acceleration voltage.

### 2.4. Western Blot Analysis

Precipitated EVs were lysed with 75 µL of RIPA buffer, and the protein concentrations were measured by the method of Bradford. A total of 20 µg of protein was separated on 12% or 15% sodium dodecyl sulphate (SDS) polyacrylamide gels and blotted on nitrocellulose membranes. Membranes were blocked with 5% low fat milk for one hour and incubated with rabbit anti-CD9 antibody (1:500; ab92726, Abcam, Cambridge, UK), mouse anti-CD63 antibody (1: 1000; MABF2159, Sigma-Aldrich, Taufkirchen, Germany), rabbit anti-syntenin-1 antibody (1:1000; ab133267, Abcam, Cambridge, UK), and rabbit anti-calreticulin antibody (1:1000 Cell Signaling Technology, Danvers, MA, USA) in 1% BSA/TBST overnight at 4 °C. For detection, a peroxidase conjugated goat anti-mouse or goat anti-rabbit antibody (Jackson Immuno Research, Suffolk, UK) were used, and protein bands were visualized with ECL chemiluminescence detection (Millipore, Billerica, MA, USA).

### 2.5. mRNA Isolation, cDNA Syntheses and qPCR Analyses

Pelleted EVs were lysed in 500 µL of TRI Reagent^®^ (Sigma-Aldrich, Taufkirchen, Germany), and 75 µL of chloroform (CarlRoth, Karlsruhe, Germany) was added, vortexed, incubated at room temperature for 5 min, and centrifuged at 10,000× *g* at 4 °C for 20 min. RNA was purified using the RNeasy Mini Elute Spin Columns (Qiagen, Hilden, Germany) according to the manufacturer’s protocols, eluted in 14 µL of RNAse-free H_2_O and stored at −80 °C. RNA concentrations were determined by NanoDrop One analyses (ThermoFisher, Darmstadt, Germany), a PCR for genomic DNA contamination was conducted with 1 µL of isolated RNA, and cDNA syntheses were performed by SuperScriptTM IV reverse transcriptase and random hexamer oligos (ThermoFisher, Darmstadt, Germany) according to the manufacturer’s protocols using the entire volume of the remaining total RNA (12 µL). Quantitative RT-PCRs were performed with a LightCycler^®^ 480 System (Roche, Mannheim, Germany) using SYBR^®^ Green JumpStart™ Taq ReadyMix (Sigma-Aldrich, Taufkirchen, Germany) with 1 µL of cDNA and 14 µL of reaction mixture in duplicate. Primers used for quantitative RT-PCR are shown in Table 1. Normalization was carried out for GAPDH (Glyceraldehyde 3-phosphate dehydrogenase). Recently, GAPDH was identified as a stable reference gene in mRNAs isolated from exosomes [35]. All PCR products were validated for a single band at an agarose gel to confirm the accuracy of the desired qPCR.

### 2.6. Nanoparticle Tracking Analysis (NTA)

NTA measurements were conducted using a NanoSight LM10 (NanoSight, Amesbury, UK), equipped with a 532 nm laser. Before acquisition, isolated EVs were resuspended and further diluted in PBS to a final concentration of about 10^8^–10^9^ EVs/mL. The measurements were performed at 25 °C. Each sample was measured at five different positions for 60 s in three independent experiments. The software NTA 3.0 was applied for capturing and analyzing the data.

### 2.7. Statistical Analyses

Data analyses were performed using GraphPad Prism 9 (La Jolla, CA, USA) and IBM SPSS Statistics software version 24 (SPSS, Chicago, IL, USA). Sample groups were tested for normal distribution. Sample groups, which were not normal distributed, were analyzed using the Mann–Whitney test or the Kruskal–Wallis test with Dunn’s multiple comparisons test. The statistical analysis methods are displayed in the corresponding figure legends. Receiver operator characteristic (ROC) was carried out with GraphPad Prism 9 (La Jolla, CA, USA), calculating the area under the curve (AUC), sensitivity, and specificity. Only AUCs higher than 0.8 were displayed and further analyzed.

## 3. Results

### 3.1. Study Population

In total, 87 gastric adenocarcinoma patients and 14 patients with Barrett’s metaplasia, who were treated in the Department of Visceral, Transplant, Thoracic and Vascular Surgery of the University Hospital of Leipzig, were included in this study. The demographic and pathological characterizations of all patients are shown in Table 2. Of these, 60 patients were recruited at their first diagnosis and had not received any treatment before (untreated GC patients = uGCP). The remaining 27 patients were recruited after their first diagnosis and had undergone various treatment algorithms, including chemotherapy and gastrectomy, in the past (treated GC patients = tGCP).

The mean age was 64 and 60 years for uGCP and tGCP, respectively, and 67 for the control group. A total of 82.8% of all cancer patients were males. The majority of the investigated patients had advanced tumors with T3–T4 (67.8%), 59.8% were classified as nodal-positive, and 59.8% had distant metastases, resulting in UICC-stages III and IV in 80.5%.

### 3.2. EV Characterization

**Figure 1:**
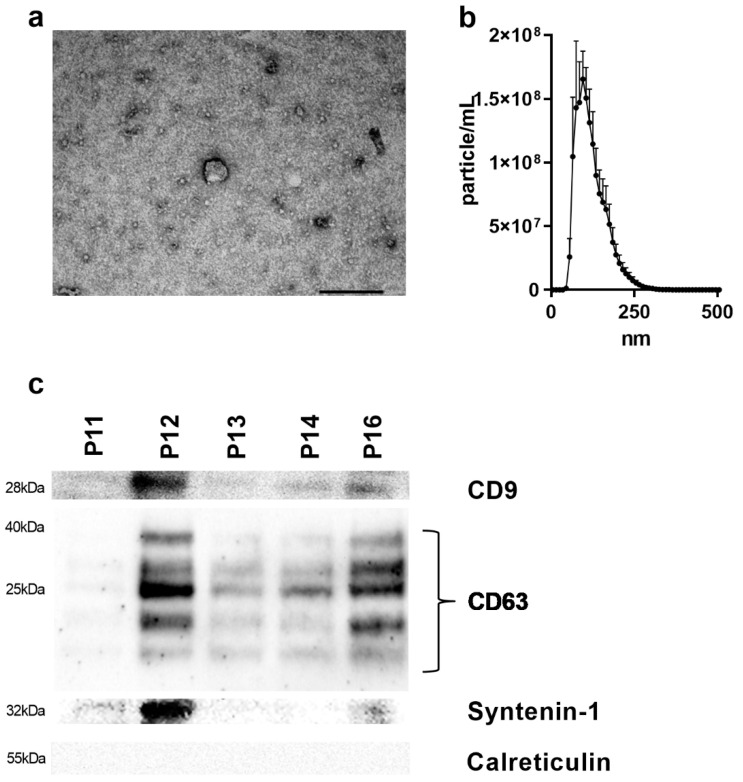
EV characterization of gastric cancer patients: (**a**) Visualization of plasma-derived EVs using TEM (scale bar; 100 nm). (**b**) Representative analysis of the particle size from plasma EVs of gastric cancer patients by NTA (*n* = 3). (**c**) Western blot analysis of CD9, CD63, syntenin-1, and calreticulin in EVs from GC patients (EV—extracellular vesicle; kDa—kilo Dalton; nm—nanometer; TEM—transmission electron microscopy).

EV isolations were possible for all 101 investigated cases. The presence of EVs was confirmed by TEM, which visualizes particles in the size range of exosomes of approximately 100 nm (Figure 1a). Furthermore, four representative samples were measured by NTA, confirming a modal size of 90 nm (range; 79–120 nm) (Figure 1b). The presence of an exosomal surface marker was validated by Western blot analysis for CD9, CD63, syntenin-1 and calreticulin. While EVs were positive for CD9, CD63, and syntenin-1, they did not contain calreticulin, a negative marker for exosomes (Figure 1c and Figures S1–S4).

### 3.3. FASN, PTEN, and CD44 mRNA Analyses

**Figure 2:**
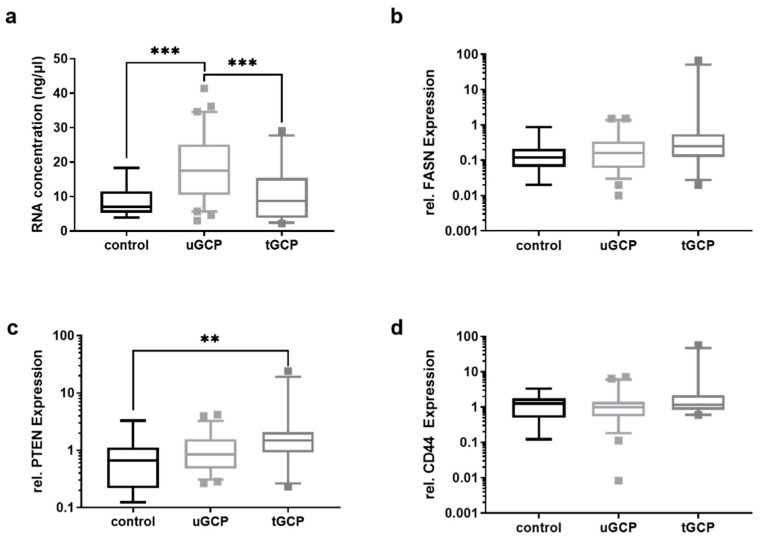
Total RNA, FASN, PTEN, and CD44 mRNA levels in controls, uGCP and tGCP. (**a**) The total RNA from EVs precipitated from 250 of µL plasma was measured, showing decreased concentrations in the tGCP cohort. The relative mRNA levels of (**b**) FASN (**c**) PTEN, and (**d**) CD44 were measured by qRT-PCR. While the levels of PTEN and FASN were higher in the tGCP cohort, the CD44 level was not altered between the uGCP and the tGCP cohort. Kruskal–Wallis test with Dunn’s multiple comparisons test was used to analyze the differences between the two groups (*** = *p* < 0.0001; n_control_ = 14; n_uGCP_ = 60; n_tGCP_ = 27; ** = *p* < 0.01; tGCP—treated GC patients; uGCP—untreated GC patients).

The amounts of total RNA from EVs, isolated from 250 μL of plasma, were significantly (*p* < 0.001) higher in uGCP, who had not received any treatment, than in patients who had already undergone chemotherapy or gastrectomy (tGCP) and the control group (Figure 2a). None of the measured RNA concentrations were under the dialectical limit of 1.6 of ng/µL. To discriminate between controls and uGCP based on the total RNA levels, a receiver operator characteristic (ROC) was performed, which revealed an AUC of 0.8286 (standard error: 0.05598; 95% CI: 0.7189 to 0.9383; *p* < 0.001), a sensitivity of 75.0% (95% CI: 62.8% to 84.2%), and a specificity of 78.8% (95% CI: 52.4% to 92.4%) (Figure S5). The relative mRNA levels of FASN, PTEN, and CD44 were analyzed using the relative mRNA levels of the reference gene GAPDH, whereby the level pattern of PTEN mRNA was significantly higher in the tGCP than in the control group (*p* = 0.0089) (Figure 2c and Figure S5b). There was no difference in the mRNA levels of FASN and CD44 between the control group, uGCP, and tGCP. All remaining ROC had shown an AUC < 0.8 (data not shown).

**Figure 3:**
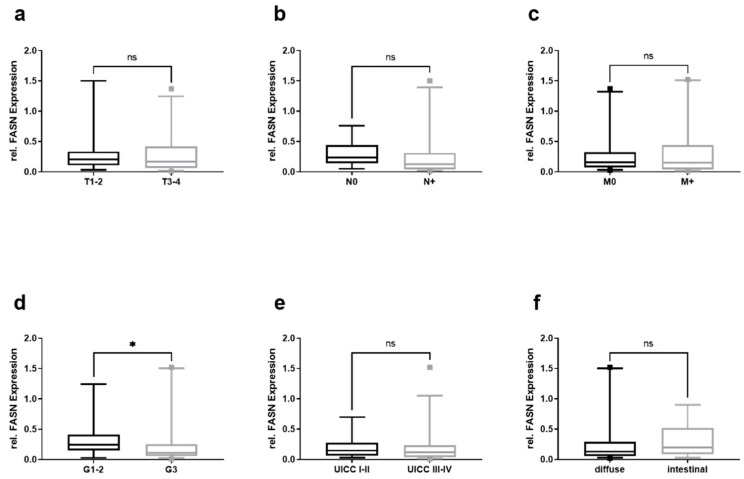
Relative FASN mRNA levels in EVs from GC patients and their clinico-pathological stratification. Relative mRNA levels of FASN in (**a**) T1–2 and T3–4 gastric cancers, (**b**) nodal negative (N0), and nodal positive (N+), (**c**) non-metastasized (M0) and metastasized (M+), (**d**) grade 1–2 (G1–2) and grade 3–4 (G3–4), (**e**) UICC-stage I–II and III–IV, and (**f**) according to Laurén’s classification. Mann–Whitney test was used to analyze the differences between the two groups (* = *p* < 0.05; G—grading; M—metastasis; n—nodal; ns – not significant; T—tumor; UICC—Union Internationale Contre le Cancer).

Furthermore, the values for uGCP were stratified according to the TNM classification, tumor grading, UICC stage, and Laurén’s classification, with significantly lower values for the FASN mRNA in patients with tumors grading G3 as compared to patients with G1–2 (Figure 3d). However, those in the T (Figure 3a), N (Figure 3b), and M category (Figure 3c) of the UICC stage (Figure 3e) and Laurén’s classification (Figure 3f) were not significantly different between the groups.

**Figure 4:**
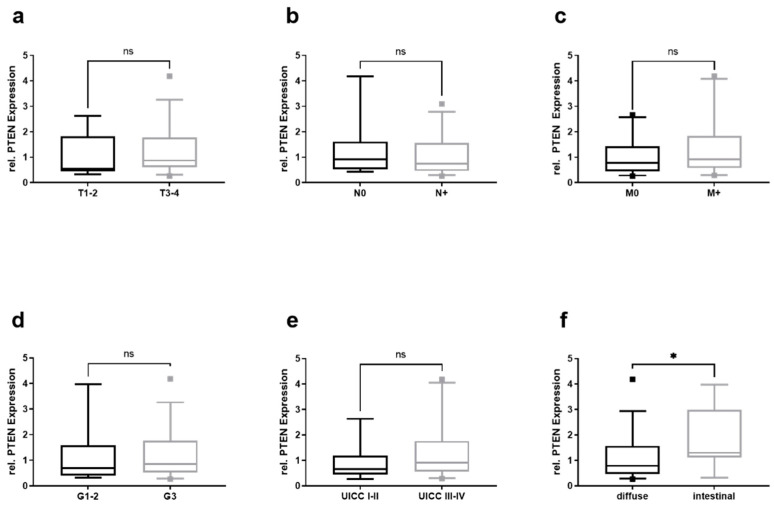
Relative PTEN mRNA levels in EVs from GC patients and their clinico-pathological stratification. Total RNA amount in (**a**) T1–2 and T3–4 gastric cancers, (**b**) nodal negative (N0) and nodal positive (N+), (**c**) non-metastasized (M0) and metastasized (M+), (**d**) grade 1–2 (G1–2) and grade 3–4 (G3–4), (**e**) UICC-stage I–II and III–IV, and (**f**) according to Laurén’s classification. Mann–Whitney test was used to analyze the differences between the two groups. (* = *p* < 0.05; G—grading; M—metastasis; n—nodal; ns – not significant; T—tumor; UICC—Union Internationale Contre le Cancer).

Relative PTEN mRNA levels were significantly higher in patients with intestinal- rather than diffuse-type gastric cancer according to Laurén’s classification (Figure 4f). The stratification for the total amount of isolated RNA (Figure S6) and CD44 (Figure S7) did not reveal any significant alteration.

### 3.4. FASN, PTEN, and CD44 mRNA Levels after Neoadjuvant Chemotherapy and Curatively Intended Gastrectomy

Additionally, matched uGCP were chosen to measure the FASN, PTEN, and CD44 mRNA levels in a longitudinal study to provide some evidence for the concept in a small confirmatory study population, which will be increased in future longitudinal cohorts. The first analysis was carried out before chemotherapy induction and the second after chemotherapy completion. The patients received FLOT (81.8%), EOX (9.1%), and CROSS (9.1%). Blood was taken after 7.18 ± 0.36 weeks after the last chemotherapy cycle. A third sample was collected 10 days after curative intended gastrectomy.

**Figure 5:**
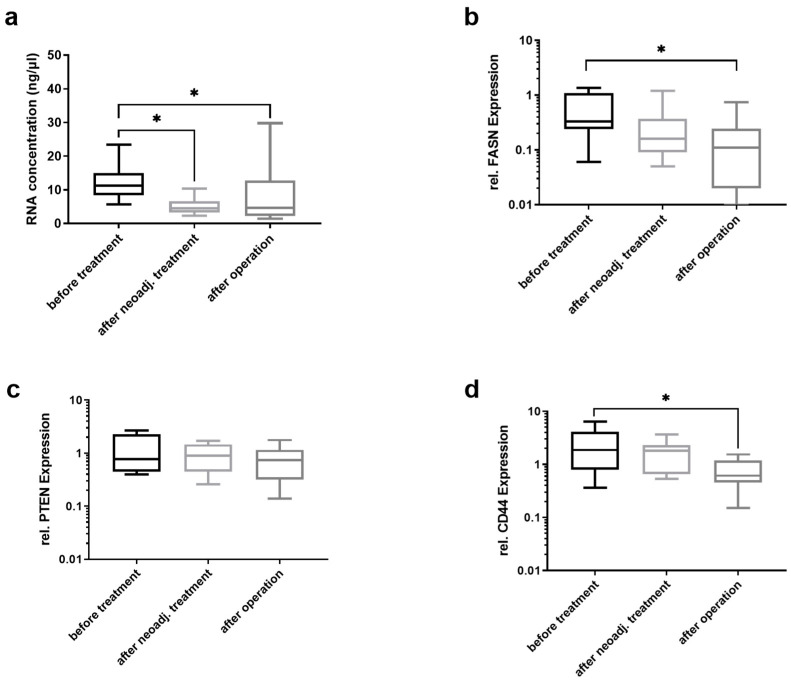
FASN, PTEN, and CD44d mRNA levels after neoadjuvant chemotherapy and curatively intended gastrectomy. In twelve patients, (**a**) the total RNA amount and the relative mRNA levels of (**b**) FASN, (**c**) PTEN, and (**d**) CD44 from EVs were measured using a Nanodrop device and by qRT-PCR. The total RNA amount and the relative mRNA levels of FASN decreased after neadjuvant therapy, and CD44 mRNA levels decreased after neodjuvant therapy as well as at 10 days post-surgery. Kruskal–Wallis test with Dunn’s multiple comparisons test was used to analyze the differences between the different time points (*n* = 11; * = *p* < 0.05).

The amount of total RNA isolated from EVs from 250 µL of plasma significantly decreased after chemotherapy and surgery (Figure 5a). However, this amount did not differ between chemotherapy completion and after gastrectomy. All patients received a complete (R0) resection. One patient had a complete (grade 1) PRR, four patients had a PRR of grade 2, and six patients had a PRR of grade 3 according to Werner and Höfler [34]. The mRNA levels of FASN (Figure 5b) decreased after surgery but not after neoadjuvant chemotherapy. PTEN mRNA levels (Figure 5c) were not significantly altered after neoadjuvant chemotherapy or surgery. The mRNA levels of CD44 were significantly lower after gastrectomy (Figure 5d).

**Figure 6:**
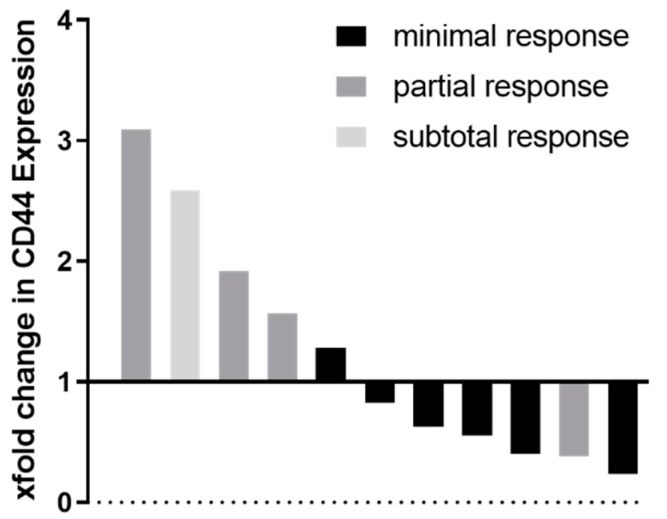
Fold change in CD44 mRNA levels in individual patients after neoadjuvant chemotherapy relative to baseline before treatment, grouped by histo-pathological response grading according to Werner and Höfler [34].

A drop in CD44 mRNA levels after neoadjuvant therapy might be associated with a poor response to therapy. The highest loss in CD44 mRNA levels was measured in patients with subtotal and partial pathological response rates (Figure 6).

## 4. Discussion

In this study, we clearly showed the suitability of total RNA isolation from plasma-derived EVs in GC patients. Moreover, the total RNA concentration and the mRNA levels of FASN, PTEN, and CD44 were analyzed in uGCP and tGCP. The total RNA and PTEN mRNA levels were found to be increased in GC patients compared to individuals not suffering from cancer. Furthermore, the mRNA levels were lower in G3 than in G1/2 tumors for FASN and decreased after neoadjuvant chemotherapy together with gastrectomy for FASN and CD44.

FASN was found to be increased using immunohistochemistry in a cohort of 60 GC patients. The height of FASN expression is variable and dependent on the TNM stage [17]. The increased FASN mRNA expression in patients with poorly differentiated GC reflects the increased FASN expression and reduced overall survival in peritoneal metastasized patients [17]. Therefore, FASN seems to be highly associated with dissemination processes and tumor aggressiveness, while tumor cells are highly dependent on the “de novo” synthesis of fatty acids by FASN [36]. The role of FASN as a direct tumor oncogene has not been well understood so far, as it promotes tumor cell growth in breast and nasopharyngeal cancer [37,38]. As FASN expression was observed in 90% of all patients with GC, it has been supposed as a diagnostic characteristic of GC cells [17]. In our cohort, the FASN mRNA level in EVs was lower in poorly differentiated than in well- and intermediate-differentiated tumors. Here, the regulation of EV secretion and active transport mechanism in the constitution of EV content might be an explanation. Furthermore, the differences in the FASN mRNA levels were only significantly altered between diagnosis and after surgery, not between post-chemotherapy and post-surgery. The drop in FASN mRNA levels after neodjuvant chemotherapy was visible, and a further drop after surgery to the levels of the control group will potentially require longer time intervals, as in our study the post-surgery measurement was performed 10 days after gastrectomy. The limitations of this study include the selection of specific mRNAs based on recent TCGA analyzes. The accurate quantification of small amounts of RNA is exacting, and there are different technical methods such as spectroscopy (e.g., NanoDrop), fluorometry (e.g., Qubit), and fragment analyses (e.g., Bioanalyzer). In this study a spectroscopic method was used, and none of the analyzed samples had a RNA concentration lower 1.6 ng/µL, which is the limit of detection of the used NanoDrop One device. Additionally, there are several methods for EV isolation and characterization. In this study, a precipitation method was used. Other methods include ultracentrifugation and column-based methods, which use antibodies against EV surface markers. The outcome of this study might be also influenced by the heterogeneity of the study population. However, our study represents a real-world patient cohort with various tumor stages, and we included a control group, which was clinically evaluated to exclude a gastrointestinal malignancy.

While CD44 was found to be amplified, the tumor suppressor PTEN was deleted in a majority of GC patients [11,14]. PTEN antagonizes the PI3K/Akt-signaling pathway, and a deletion leads to uncontrolled PI3K/Akt-activation [11,15]. Furthermore, there was a lack in PTEN expression correlated with metastasis and tumor invasion and with diffuse type according to Laurén’s classification [16]. This fits well with the lower PTEN mRNA levels we found in diffuse type tumors, reflecting the potential use of PTEN mRNA levels from EVs as a diagnostic marker. The slightly higher FASN mRNA levels in the tGCP cohort compared to the control group might be explained by the more advanced disease stage of those patients, who had already received several chemotherapy cycles and/or pressurized intraperitoneal aerosol chemotherapy (PIPAC) in palliative intent in order to decrease associated symptoms. The higher decrease in the CD44 mRNA levels after gastrectomy compared to the completion of the perioperative chemotherapy is in line with the curative intended surgery and the moderate PRR observed in these patients (median stage: 2.5 according to Werner and Höfler) [34]. Apart from mRNAs, EVs also contain sufficient amounts of miRNAs, and a panel of four miRNAs (miR-19b-3p, miR-17-5p, miR-30a-5p, and miR-106a-5p) was used to discriminate between GC patients and healthy controls [39]. Circulating miRNAs were also analyzed cell- and vesicle-free. However, EV-derived miRNAs are more stable and might have bioactive capacity, as they act as a delivery system of tumor cell components [40]. Additionally, two other studies also described cell-free miRNA (miRNA-199a-3p and miRNA-21) to be altered in GC patients [41,42]. Traditionally, specific surface molecules, such as CD9, CD63 and CD81, characterize EVs and exosomes. Recently, syntenin-1 was discovered as a new, more reliable EV marker than CD9, CD63, and CD81 [32]. To discover specific EVs from organs or tumors, an individual and potentially exclusive EV marker needs to be identified. Decreased QSOX1 protein levels in exosomes from patients with colorectal cancer have been recently identified, which might serve as an indicator for malignant transformation in CRC [43]. Glycipan-1 was shown to identify pancreatic cancer-derived exosomes, while serum-derived exosomes from patients with a primary gastro-esophageal adenocarcinoma were characterized by a loss of glycipan-3 [44,45]. Regardless the investigated exosomal cargo (mRNA, microRNA, proteins), further prospective trials are necessary for validation of a clinical use of the non-invasive diagnostic by EVs and exosomes.

The prediction of chemo-response is crucial, and GC patients would benefit from it at the very beginning of diagnosis. Recently, germline polymorphisms were discovered to potentially be associated with a significant better long-term survival in the perioperative CTx arm of the MAGIC-trial. However, whether this retrospective biomarker has any predictive potential to forecast chemo-response is uncertain [46]. Taking into account that the total mRNA amount and the relative mRNA levels of FASN and CD44 decreased after the neoadjuvant chemotherapy and gastrectomy, stratification with regard to the pathological response rate for therapy success of neoadjuvant chemotherapy needs further prospective studies. Nevertheless, all GC patients received a platinum- and 5-FU-based chemotherapy for their treatment on a regular basis [47]. With regard to the molecular biology of GC, only trastuzumab is currently widely available as a targeted therapy option [48]. Others, such as FGFR2, EGFR, PARP, STAT3, and two recently described immunotherapy agents, had negative results [47,49]. Even the frequency of these molecular targets is lesser than HER2-amplification [11].

## 5. Conclusions

Liquid biopsies will offer a new era in the diagnosis and monitoring of disease development. Despite advances in diagnostics and treatment, the overall survival for advanced GC patients remains poor. Nodal as well as peritoneal dissemination is a common phenomenon, as 10–40% of GC patients show up with synchronous peritoneal metastases at initial diagnosis [50,51,52]. Curation in metastasized GC patients is difficult to achieve, and novel molecular biomarkers might be needed to overcome this clinically relevant diagnostic and therapeutic gap. Eventually, stratification according to the molecular subtypes in GC will predict a personalized oncologic therapy, as the concept of multimodal treatment, as an individualized overall and progression-free survival for the four molecular subtypes of GC has been shown [13]. For MSI-high gastric tumors, the usage of neoadjuvant treatment is currently discussed with controversy, as these patients do not seem to benefit from a classical platinum- and 5-FU-based chemotherapy [53,54]. However, MSI could be a new biomarker for identifying GC patients, which will respond from immunotherapy.

Confirmations of biomarkers, such as EVs, are urgently needed in further large-scale prospective studies in order to ensure the personalized treatment stratification of cancer patients. Thereby, liquid biopsies, as a less invasive tool, will play an immanent role in diagnosis, treatment monitoring, and response prediction. To achieve these issues, looking beyond GC, further prospective multicenter clinical trials are needed with a focus on standardized pre-processing and pre-analytical properties, EV separation protocols, and the analyses of reliable biomolecules, including medical and biology experts from the individual fields [33]. Our data have clearly shown that total RNA isolation and characterization from EVs is feasible in a real-world GC patient cohort. The further evaluation of EV cargos might be a useful clinical criterion for the characterization of GC patients.

## Figures and Tables

**Table 1:** Oligo sequences used for qRT-PCR.

Name	Forward	Reverse	Reference	Length (BP)
GAPDH	GAGTCAACGGATTTGGTCGTA	TTCCCGTTCTCAGCCTTGAC	NM_002046	178
FASN	CAGAGCAGCCATGGAGGAG	CATCGTCCGTGACCATGTCC	NM_004104	109
CD44S	GCAGTCAACAGTCGAAGAAGG	TGTCCTCCACAGCTCCATT	NM_000610	76
PTEN	AGTGGCACTGTTGTTTCACA	CACCTTTAGCTGGCAGACCA	NM_000314	97

Bp—base pairs; CD44—cluster of differentiation 44; GAPDH—glyceraldehyde 3-phosphate dehydrogenase; FASN—fatty acid synthase; PTEN—phosphatase and tensin homolog; qRT-PCR—quantitative reverse transcription polymerase chain reaction.

**Table 2:** Clinico-pathological patients’ characteristics.

Characteristics	Number of Cases	Controls	uGCP	tGCP
Mean age [years]		67	64	60
Gender				
Male	72 (71.3%)	12 (85.7%)	41 (68.3%)	19 (70.7%)
Female	29 (28.7%)	2 (14.3%)	19 (31.7%)	8 (29.3%)
T-category				
T1	6 (6.9%)		5 (8.3%)	1 (3.7%)
T2	11 (12.6%)		6 (10.0%)	5 (18.5%)
T3	46 (52.9%)		35 (58.3%)	11 (40.7%)
T4	13 (14.9%)		5 (8.3%)	8 (29.6%)
Tx	11 (12.6%)		9 (15.0%)	2 (7.4%)
N-category				
N0	24 (27.6%)		15 (25.0%)	9 (33.3%)
N+	52 (59.8%)		37 (61.7%)	15 (55.6%)
Nx	11 (12.6%)		8 (13.3%)	3 (11.1%)
M-category				
M0	34 (39.1%)		28 (46.7%)	6 (22.2%)
M+	52 (59.7%)		31 (51.7%)	21 (77.8%)
Mx	1 (1.1%)		1 (1.7%)	0 (0.0%)
Localization				
AEG-II and -III	34 (39.1%)		26 (43.3%)	8 (29.6%)
Gastric Corpus and Antrum	53 (60.9%)		34 (56.7%)	19 (70.4%)
Grading				
G1	4 (4.6%)		2 (3.3%)	2 (7.4%)
G2	14 (16.1%)		11 (18.3%)	3 (11.1%)
G3	52 (59.8%)		38 (63.3%)	14 (51.9%)
n.a.	17 (19.5%)		9 (15.0%)	8 (29.6%)
Laurén’s-classification				
Intestinal type	16 (18.4%)		10 (16.7%)	6 (22.2%)
Diffuse type	57 (65.5%)		37 (42.5%)	20 (74.1%)
Mixed type	1 (1.1%)		1 (1.7%)	0 (0.0%)
n.a.	13 (14.9%)		12 (20%)	1 (3.7%)
UICC-stage				
I	8 (9.2%)		5 (8.3%)	3 (11.1%)
II	8 (9.2%)		7 (11.7%)	1 (3.7%)
III	15 (17.2%)		14 (23.3%)	1 (3.7%)
IV	55 (63.2%)		34 (56.7%)	22 (81.5%)

G—grading; M- metastasis; N—nodal; n.a.—not applicable; T—tumor; Tgcp—treated GC patients; uGCP—untreated GC patients; UICC—Union Internationale Contre le Cancer.

## Data Availability

The datasets generated during the current study are available from the corresponding author on reasonable request.

## Supplementary Materials

The following are available online at https://www.mdpi.com/article/10.3390/cancers13235975/s1, Figure S1: Western blot for CD63, Figure S2: Western Blot for CD9, Figure S3: Western Blot for syntenin-1, Figure S4: Western blot for calreticulin, Figure S5: ROC for total RNA between controls and uGCP, Figure S6: Total RNA levels in EVs from GC patients and their clinico-pathological stratification, Figure S7: Relative CD44 mRNA levels in EVs from GC patients and their clinico-pathological stratification.

## Abbreviations

5-FU: 5-fluorouracil; AEG: adenocarcinoma of the esophagastric junction (according to Siewert’s classification); AUC: area under the curve; CD: cluster of differentiation; CTx: chemotherapy; CROSS: 41.4Gy plus carboplatin/paclitaxel; CTCs: circulating tumor cells; EGFR: epidermal growth factor receptor; EOX: epirubicin, oxaliplatin, capecitabine; EV: extracellular vesicle; FASN: fatty acid synthase; FGFR2: fibroblast growth factor receptor 2; FLOT: 5-FU, leucovorin, oxaliplatin, docetaxel; G: grade; GC: gastric cancer; GS: genomic stable; HER2: epidermal growth factor receptor 2; Hsp: heat-shock proteins; M: metastasis; MSI: microsatellite instable; N: nodal; n.a.: not applicable; ns: not significant; NTA: nanoparticle tracking analysis; OS: overall survival; PARP: poly (ADP-ribose) polymerase; PBS: phosphate-buffered saline; PI3K/Akt: phosphoinositide 3-kinases; PIPAC: pressurized intraperitoneal aerosol chemotherapy; PRR: pathological response rate; PTEN: phosphatase and tensin homolog; STAT3: signal transducer and activator of transcription 3; R: resection; ROC: receiver operator characteristic; T: tumor; TEM: transmission electron microscopy; TCGA: The Cancer Genome Atlas; tGCP: treated GC patients; uGCP: untreated GC patients; UICC: Union Internationale Contre le Cancer; WHO: World Health Organization.
