# Supplementary material for: Characterization of Total RNA, CD44, FASN, and PTEN mRNAs from Extracellular Vesicles as Biomarkers in Gastric Cancer Patients
Source: Cancers (Basel). 2021 Nov 27;13(23):5975. doi: 10.3390/cancers13235975 (PMC8656496; doi:10.3390/cancers13235975)
Supplement: Supplementary file 1 [file cancers-13-05975-s001.zip › cancers-1476463-supplementary.pdf]

Figure S1

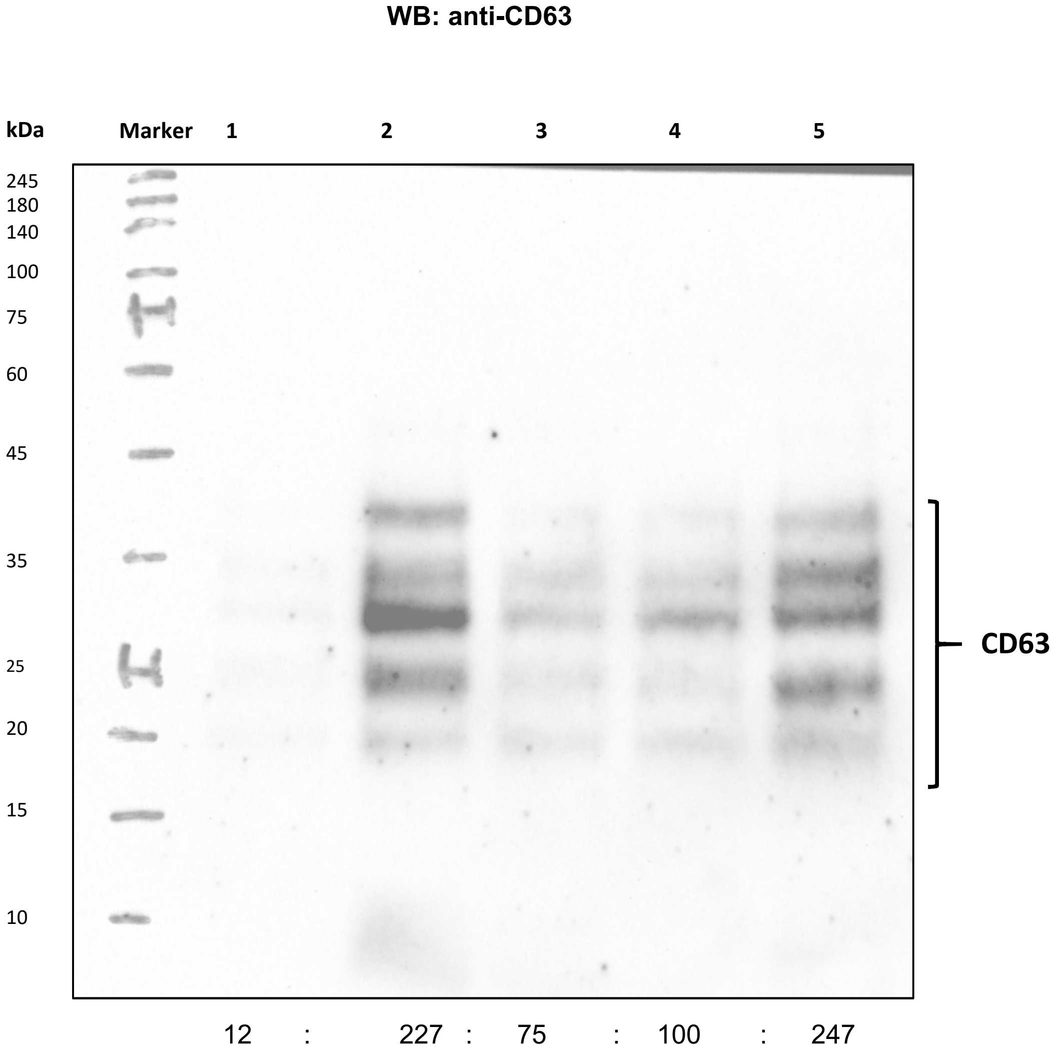

WB: anti-CD63
kDa
Marker
1
2
3
4
5
245
180
140
100
75
60
45
35
25
20
15
10
CD63
12 : 227 : 75 : 100 : 247

Figure S2

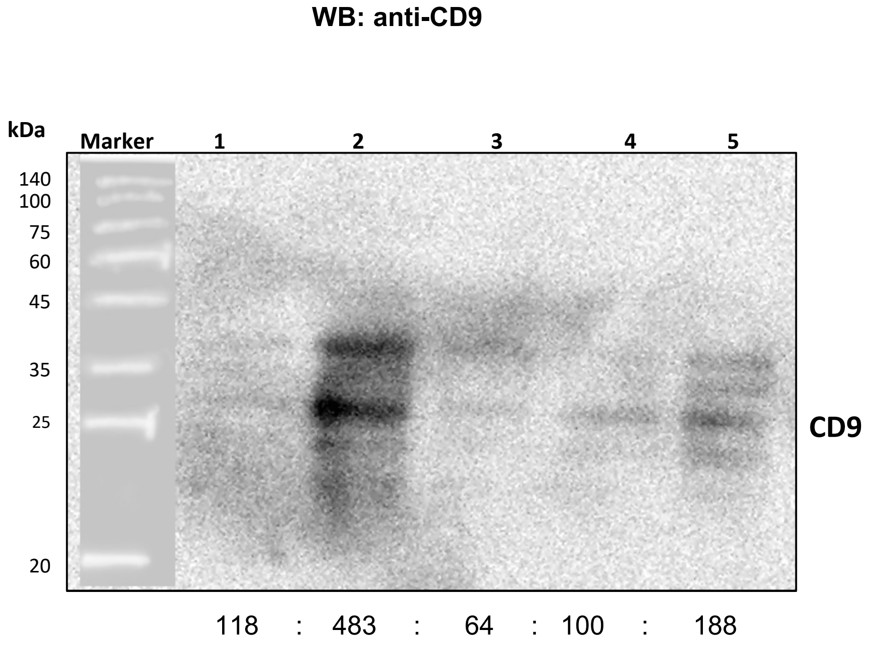

WB: anti-CD9
kDa
Marker
1
2
3
4
5
140
100
75
60
45
35
25
20
CD9
118 : 483 : 64 : 100 : 188

Figure S3

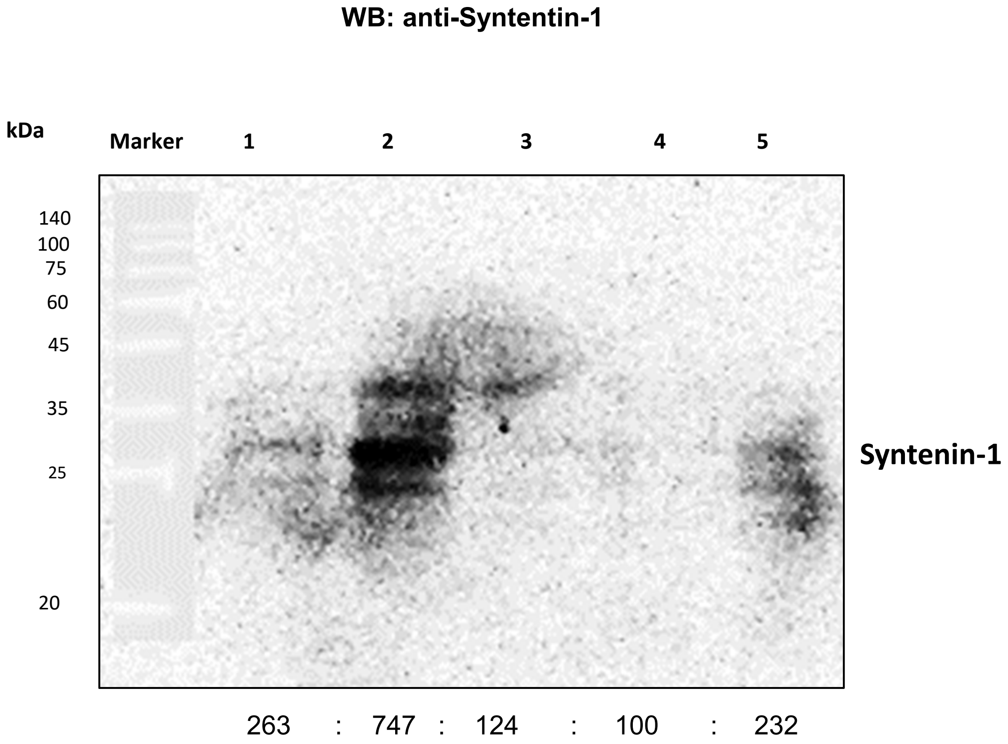
WB: anti-Syntentin-1
kDa
Marker
1
2
3
4
5
140
100
75
60
45
35
25
20
Syntenin-1
263 : 747 : 124 : 100 : 232

Figure S4

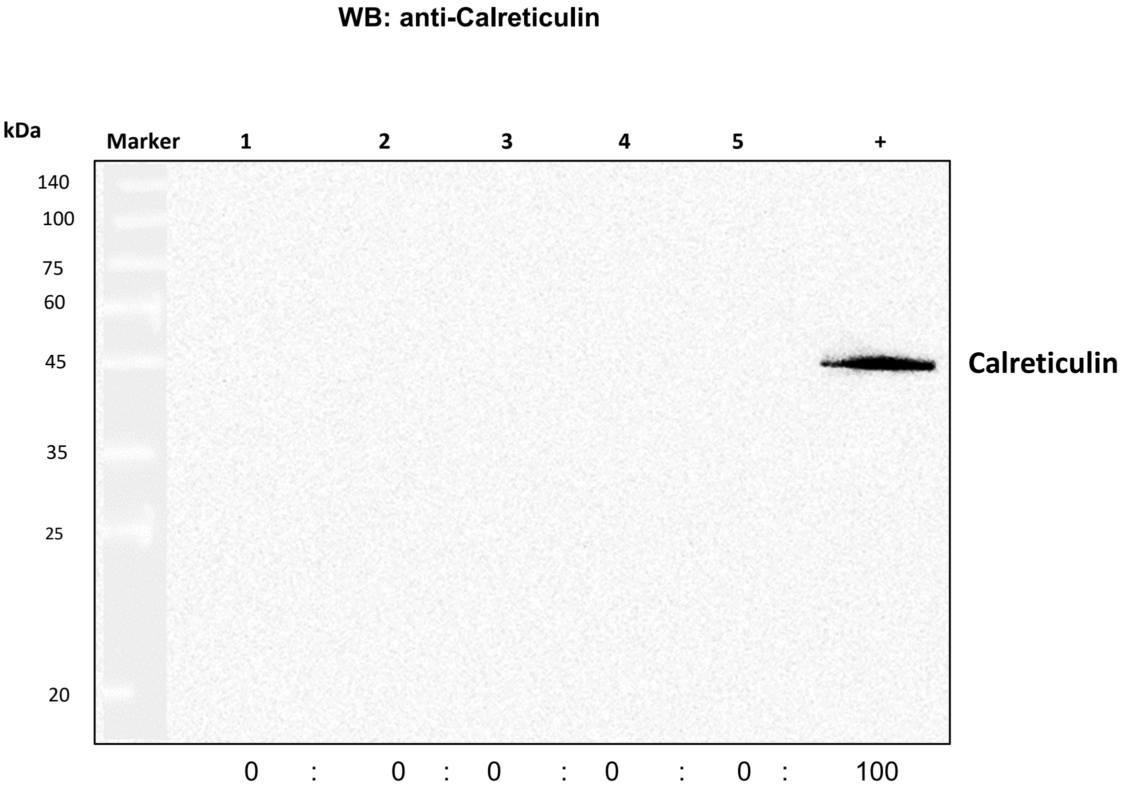
WB: anti-Calreticulin
kDa
Marker
1
2
3
4
5
+
140
100
75
60
45
35
25
20
Calreticulin
0 : 0 : 0 : 0 : 0 : 100

Figure S5

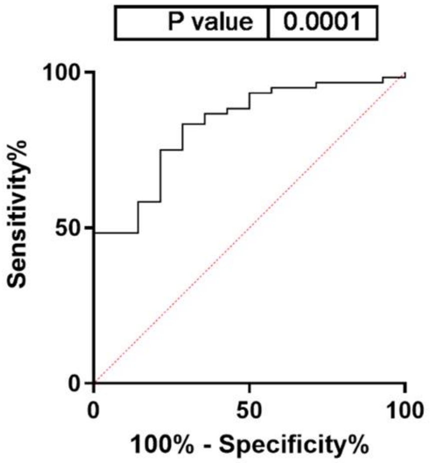

AUC: 0.8286 (standard error: 0.05598; 95% CI: 0.7189 to 0.9383; p<0.001); Sensitivity: 75.0% (95% CI: 62.8% to 84.2%); Specificity: 78.8% (95% CI: 52.4% to 92.4%)

Figure S6

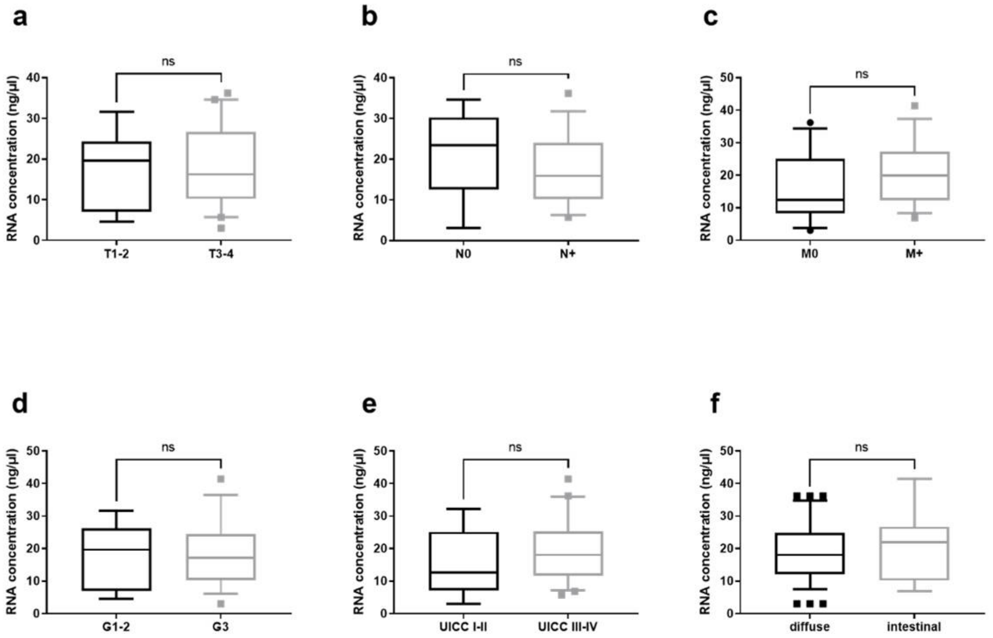

Figure S6. Total RNA levels in EVs from GC patients and their clinico-pathological stratification. Total RNA levels in (a) T1-2 and T3-4 gastric cancers, (b) nodal negative (N0), and nodal positive (N+), (c) non-metastasized (M0) and metastasized (M+), (d) grade 1-2 (G1-2) and grade 3-4 (G3-4), (e) UICC-stage I-II and III-IV, and (f) according to the Laurén's classification. Mann Whitney-test was used to analyze the differences between the two groups (G – grading; M – metastasis; n – nodal; ns – not significant; T – tumor; UICC - Union Internationale Contre le Cancer).

Figure S7

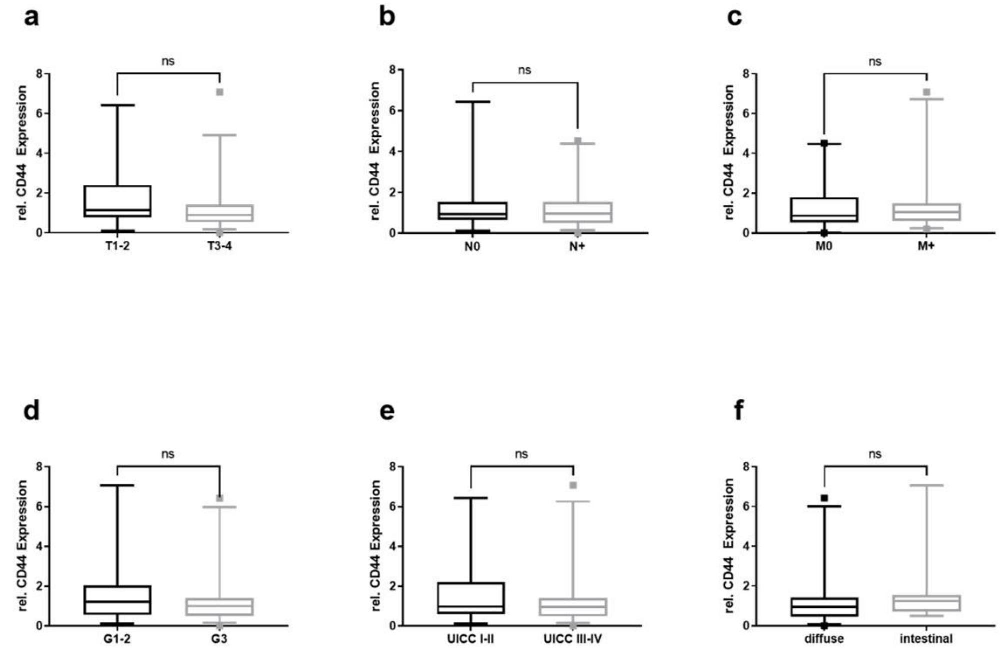

Figure S7. Relative CD44 mRNA levels in EVs from GC patients and their clinico-pathological stratification. Relative mRNA levels of CD44 in (a) T1-2 and T3-4 gastric cancers, (b) nodal nega-tive (N0), and nodal positive (N+), (c) non-metastasized (M0) and metastasized (M+), (d) grade 1-2 (G1-2) and grade 3-4 (G3-4), (e) UICC-stage I-II and III-IV, and (f) according to the Laurén's classification. Mann Whitney-test was used to analyze the differences between the two groups (G – grading; M – metastasis; n – nodal; ns – not significant; T – tumor; UICC - Union Internationale Contre le Cancer).
